# Dissecting Epstein-Barr Virus-Specific T-Cell Responses After Allogeneic EBV-Specific T-Cell Transfer for Central Nervous System Posttransplant Lymphoproliferative Disease

**DOI:** 10.3389/fimmu.2018.01475

**Published:** 2018-06-27

**Authors:** Rebecca E. Schultze-Florey, Sabine Tischer, Leonie Kuhlmann, Patrick Hundsdoerfer, Arend Koch, Ioannis Anagnostopoulos, Sarina Ravens, Lilia Goudeva, Christian Schultze-Florey, Christian Koenecke, Rainer Blasczyk, Ulrike Koehl, Hans-Gert Heuft, Immo Prinz, Britta Eiz-Vesper, Britta Maecker-Kolhoff

**Affiliations:** ^1^Pediatric Hematology and Oncology, Hannover Medical School, Hannover, Germany; ^2^Integrated Research and Treatment Center Transplantation (IFB-Tx), Hannover Medical School, Hannover, Germany; ^3^Hannover Medical School, Institute for Transfusion Medicine, Hannover, Germany; ^4^Hannover Medical School, Institute of Immunology, Hannover, Germany; ^5^Department of Pediatric Hematology and Oncology, Berlin Institute of Health, Charité – Universitätsmedizin Berlin, Freie Universität Berlin, Berlin, Germany; ^6^Department of Neuropathology, Berlin Institute of Health, Charité – Universitätsmedizin Berlin, Freie Universität Berlin, Berlin, Germany; ^7^Department of Pathology, Berlin Institute of Health, Charité – Universitätsmedizin Berlin, Freie Universität Berlin, Berlin, Germany; ^8^Department of Hematology, Oncology, Hemostaseology, and Stem Cell Transplantation, Hannover Medical School, Hannover, Germany; ^9^Hannover Medical School, Institute of Cellular Therapeutics, Hannover, Germany

**Keywords:** posttransplant lymphoproliferative disease, adoptive T cell therapy, T cell receptor sequencing, transplantation, Epstein–Barr virus

## Abstract

Epstein–Barr virus (EBV)-associated posttransplant lymphoproliferative disease (PTLD) with central nervous system (CNS) involvement is a severe complication after solid organ transplantation. Standard treatment with reduction of immunosuppression and anti-CD20 antibody application often fails leading to poor outcome. Here, we report the case of an 11-year-old boy with multilocular EBV-positive CNS PTLD 10 years after liver transplantation. Complete remission was achieved by repeated intravenous and intrathecal anti-CD20 antibody rituximab administration combined with intrathecal chemotherapy (methotrexate, cytarabine, prednisone) over a time period of 3 months. Due to the poor prognosis of CNS PTLD and lack of EBV-specific T-cells (EBV-CTLs) in patient’s blood, we decided to perform EBV-directed T-cell immunotherapy as a consolidating treatment. The patient received five infusions of allogeneic EBV-CTLs from a 5/10 HLA-matched unrelated third-party donor. No relevant acute toxicity was observed. EBV-CTLs became detectable after first injection and increased during the treatment course. Next-generation sequencing (NGS) TCR-profiling verified the persistence and expansion of donor-derived EBV-specific clones. After two transfers, epitope spreading to unrelated EBV antigens occurred suggesting onset of endogenous T-cell production, which was supported by detection of recipient-derived clones in NGS TCR-profiling. Continuous complete remission was confirmed 27 months after initial diagnosis.

## Introduction

Posttransplant lymphoproliferative disease (PTLD) constitutes a heterogeneous group of lymphoproliferative disorders occurring as severe complications of immunosuppression after solid organ transplantation (SOT). Acquired by up to 15% of pediatric transplant recipients, most cases of childhood PTLD are of B-cell origin and associated with Epstein–Barr virus (EBV) infection or reactivation (1, 2). Long-lasting immunosuppressive therapy to prevent graft rejection as well as lack of EBV-specific immunity at the time of transplantation contribute to the high incidence and unfavorable prognosis of PTLD in children (1). Up to 20% of affected patients eventually succumb to the disease (1). While modulation of immunosuppressive therapy may be sufficient in some patients, multi-agent immuno-/chemotherapy serves as the primary treatment option for advanced stage PTLD in children resulting in 80% overall survival (3). In the PTLD-1 study, complete response to Rituximab conferred a favorable outcome in adults (4). Central nervous system (CNS) PTLD displays an unfavorable outcome with 30–50% overall survival (isolated disease) (5–7) and as low as 0–10% (combined systemic and CNS disease) (7, 8). For these high-risk patients, very limited treatment options are available. Intrathecal rituximab as a combination to intravenous immuno-/chemotherapy is a promising treatment option (9). In addition, transfer of EBV-specific T-cell lines manufactured from healthy volunteers has shown promise in some patients with CNS involvement (10). Here, we report the first case of treatment of an SOT patient with CNS PTLD receiving freshly isolated, partially HLA-matched EBV-specific T-cells (EBV-CTLs) from an unrelated third party donor in addition to intravenous and intrathecal chemo-/immunotherapy.

## Methods

### Ethical Approval and Patient Informed Consent

The study was approved by the IRB of Hannover Medical School. The patient’s legal guardian gave written informed consent to both participation in the research project and publication of the case report.

### Donor Pre-Testing, Production of EBV-CTLs, and Application

Frequencies of EBV-CTLs were determined in patient mother’s blood (not sufficient for transfer) as well as in five partially HLA-matched potential donors from the alloCELL T-cell donor registry (www.alloCELL.org, Tables 1 and 2) as described using EBV peptide pools EBV nuclear antigen 1 (ppEBNA-1) and EBV Select (ppSelect) (Miltenyi Biotec, Bergisch-Gladbach, Germany) (11).

**Table 1:** Donor selection: HLA characteristics and verification of donor’s Epstein–Barr virus (EBV)-specific memory T cells.

	Donor type	HLA-type					HLA match	IFN-γ EliSpot (spw/2.5 × 10^5^ PBMCs)			IFN-γ CSA [% IFN-γ CD3^**+**^ T cells]	
		
		HLA-A	HLA-B	HLA-C	HLA-DR	HLA-DQ		EBNA1	Select	EBNA1 + Select	EBNA1 + Select	
											*OF*	*TCF*
Patient		03	07/14	07/08	01/15	05		0	1	/	/	/
Mother	PMRD							0	16	10	0.04	4.26
TPD 1	PMUD	03/11	07	07	15/16	05/06	5/10	23	TNTC	108	1.25	48.35
TPD 2	PMUD	03	07	07	15	06	5/10	3	8	3	0.01	5.28
TPD 3	PMUD	03	07	07	15	03/06	5/10	5	61	13	0.09	56.35
TPD 4	PMUD	03/11	07	07	15	06	4/10	35	120	114	0.04	16.36
TPD 5	PMUD	03	07/18	07	12/15	06/07	5/10	32	162	141	0.21	68.85

*For EliSpot assay and CSA, EBV-specific T cells were activated by 4 h *in vitro* restimulation with peptide pools EBNA1, Select and both in combination (EBNA1 + Consensus), respectively. TPD, third party donor; PMRD, partially matched related donor; PMUD, partially matched unrelated donor; HLA, human leukocyte antigen; IFN-γ, interferon-gamma; spw, spot per well; CSA, cytokine secretion assay; OF, original fraction, before enrichment; TCF, T-cell fraction, after magnetic enrichment; TNTC, too numerous to count*.

**Table 2:** T-cell receptor CDR3 sequences of clones displayed in Figures 3A,B.

cdr3 clones selectively detected in T cell product (donor = D) and post transfer				cdr3 clones detected in recipient (R) before transfer and post transfer			
EBNA.D1	CASSSKRQVPDTQYF	Select.Dl	CASSPRQADEQFF	EBNA.R1	CASSDDFFSHTDTQYF	Select.Rl	CASSDDFFSHTDTQYF
EBNA.D2	SSARDGDLRGQFF	Select.D2	CSVGQAAYEQYF	EBNA.R2	CASSLTGRTVTDTQYF	Select.R2	CASSLTGRTVTDTQYF
EBNA.D3	CSAPGQVQETQYF	Select.D3	CSAPGQVQETQYF	EBNA.R3	CASSRVGAANEQFF	Select.R3	CASSRVGAANEQFF
EBNA.D4	CASSFASGGSSYNEQFF	Select.D4	CASSPSGVPGANVLTF	EBNA.R4	CASSFRDRQDYEQYF	Select.R4	CATSPGVEQYF
EBNA.D5	CASSLRGTEAFF	Select.D5	CASSLLQGADTEAFF	EBNA.R5	CASSQDLAGGLLSYEQYF	Select.R5	CASSLEGPGYNEQFF
EBNA.D6	CASSLEGDRHQHF	Select.D6	CASSPVRSSETQYF	EBNA.R6	CASSNTDTQYF	Select.R6	CASNNLPGLETQYF
EBNA.D7	CASSLERDRPQHF	Select.D7	CASSLPTGGYYEQYF	EBNA.R7	CATSPGVEQYF	Select.R8	CASSPSRNTEAFF
EBNA.D8	CASSAGPATNEKLFF	Select.D8	CASSLSYEQYF	EBNA.R8	CAISKRLFSYNEQFF	Select.RS	CASSFRDRQDYEQYF
EBNA.D9	CASSTTDTQYF	Select.D9	CASNKLPGLETQYF	EBNA.R9	CSARDGDLRGQFF	Select.R9	CAISKRLFSYNEQFF
EBNA.D10	CASSQFGGNTIYF	Select.D10	CASSVRASPLHF	EBNA.RI0	CASSQDRGRSPLHF	Select.Rl0	CASSQTSGDGDTQYF
EBNA.D11	CASNVGYSRPDNEQFF	Select.D11	CASSLRTGELFF	EBNA.R11	CASRTPSGGAWETQYF	Select.Rll	CASSQDPSAEQFF
EBNA.D12	CASSLSGAYEQYF	Select.D12	CASSLVTNEQFF	EBNA.R12	CASSYRLGRLNQPQHF	Select.R12	CASSQGRDNSYEQYF
EBNA.D13	CASSLGGDRPQHF	Select.D13	CASSHQGGGQMRTGELFF	EBNA.R13	CASSSGIFNYGYTF	Select.R13	CASSGDIPTEHRDTQYF
EBNA.D14	CSAPGQVRETQYF	Select.D14	CAWRETGGEVSEQYF	EBNA.R14	CASSPSRNTEAFF	Select.R14	CASRTPSGGAWETQYF
EBNA.D15	CASSWEGDRPQHF	Select.D15	CASSPPGGGDQETQYF	EBNA.R15	CASSSGTGFQETQYF	Select.R15	CASSQDLAGGLLSYEQYF
EBNA.D16	CASSLEGDRPQHC	Select.D16	CASKRGGNTEAFF	EBNA.R16	CASSYLRIARPDYGYTF	Select.R16	CASSNTDTQYF
EBNA.D17	CSVGEQYI	Select.D17	CASSQETGSYEQYF	EBNA.R17	CAWSPGFTEAFF	Select.R17	CASSSGIFNYGYTF
E8NA.D18	CASSHDSSDEQYF	Select.D18	CASSEAVPGHQNTEAFF	EBNA.R18	CASSDPRGHEQYF	Select.R18	CASSYRLGRLNQPQHF
		Select.D19	CASSSGDEQYF	EBNA.R19	CASSEEELDNNQPQHF	Select.R19	CASSSGTGFQETQYF
		Select.D20	CASSVSEGNTIYF	EBNA.R20	CASSFETGGTGELFF	Select.R20	CSARDGDLRGQFF
		Select.D21	CASSLTGFLNTEAFF	EBNA.R21	CASSQAWYSGNTIYF	Select.R21	CASSQDRGRSPLHF
		Select.D22	CASSFSRDWNTEAFF	EBNA.R22	CSVEVENRNTEAFF	Select.R22	CASSDPRGHEQYF
		Select.D23	CAVNGGQFSGNTIYF	EBNA.R23	CASSPGQHNSPLHF	Select.R23	CAWSPGFTEAFF
		Select.D24	CASTFRMRPQDTQYF	EBNA.R24	CSARPRGQPYEQYF	Select.R24	CASSFETGGTGELFF
		Select.D25	CSAPGRVQETQYF	EBNA.R25	CASSQDPSAEQFF	Select.R25	CASSYLRIARPDYGYTF
		Select.D26	CASSRDKAYEQYF	EBNA.R26	CASNNLPGLETQYF	Select.R26	CASSPGQHNSPLHF
		Select.D27	CASTFRMLPQDAQYF	EBNA.R27	CASSIVNEAFF	Select.R27	CASSEEELDNNQPQHF
		Select.D28	CASS F PAVGLPSSSYN EQF F			Select.R28 Select.R29	CASSQAWYSGNTIYF CSARPRGQPYEQYF

Manufacturing of clinical-grade EBV-specific CD4+ and CD8+ T-cells from EBV-seropositive allogeneic 5/10 HLA-matched third party donor (TPD 1, Tables 1 and 2) was performed on a CliniMACS device using ppEBNA1 and ppSelect in combination and the IFN-γ Cytokine Capture System (Miltenyi Biotech). Quality control of the final T-cell product was done as described (11). Details on the T-cell manufacturing and product can be found in the Supplementary Material. The patient got one fresh and four cryopreserved EBV-specific T-cell products from a single manufacturing process.

### Monitoring

Monitoring of viral load and EBV-specific T-cell frequencies in patient’s blood was done before and after T-cell transfer by IFN-γ ELISpot assay as described and using the following peptide pools: ppEBNA1, ppSelect, ppLMP2a, ppBZLF1 (all Miltenyi Biotec) (12, 13). If suitable numbers of PBMCs were obtained, EBV-CTLs were expanded over 7 days using the respective antigens ppEBNA1 and ppSelect in TexMACS media (Miltenyi Biotec) containing 50 U/ml IL-2 (Peprotec). After 7 days, IFN-γ ELISpot assay was repeated using the respective antigens. Expanded cells were used for TCR beta chain repertoire analysis.

### TCR Beta Chain Repertoire Analysis

The stimulated and expanded PBMCs were stained with following antibodies: dead/alive (DAPI), hCD45^+^ (APC-Vio770), hCD3^+^ (PE-Cy7), hTCR αβ^+^ (FITC), and hCD8^+^ (VioGreen). They were sorted into CD8^+^ T-cells with a FACS Aria Fusion flow cytometer. mRNA was extracted using the RNeasy Plus Micro Kit (QIAGEN) and then reverse-transcribed into cDNA according to the SMARTer RACE 5′-3′ PCR Kit (Clontech) manual. Then, a combined amplification of the TCR β CDR3-region and Illumina adaptor sequences were performed with the Advantage 2 PCR Kit (Clontech). After a DNA sample size identifying gel electrophoresis, the bands were extracted with the Gel extraction Kit (QIAGEN). Indexing of the samples was performed with Nextera Primer Kit (Illumina) in another Advantage 2 PCR and the product was purified with the Agencourt AMPure XP Kit. The DNA concentration was measured with the Qubit 2.0 fluorometer, samples were pooled, and the pool was set to 4 nM. Denaturation and dilution of the pool was done as described at the Illumina MiSeq Dilution and Denaturation Guide. Finally, next generation sequencing (NGS) was performed on the Illumina MiSeq System. For the analysis, the FastQ files were annotated at IMGT/HighV-Quest database and processed with tcR-package and VDJtools.

## Case Presentation

**Figure 1:**
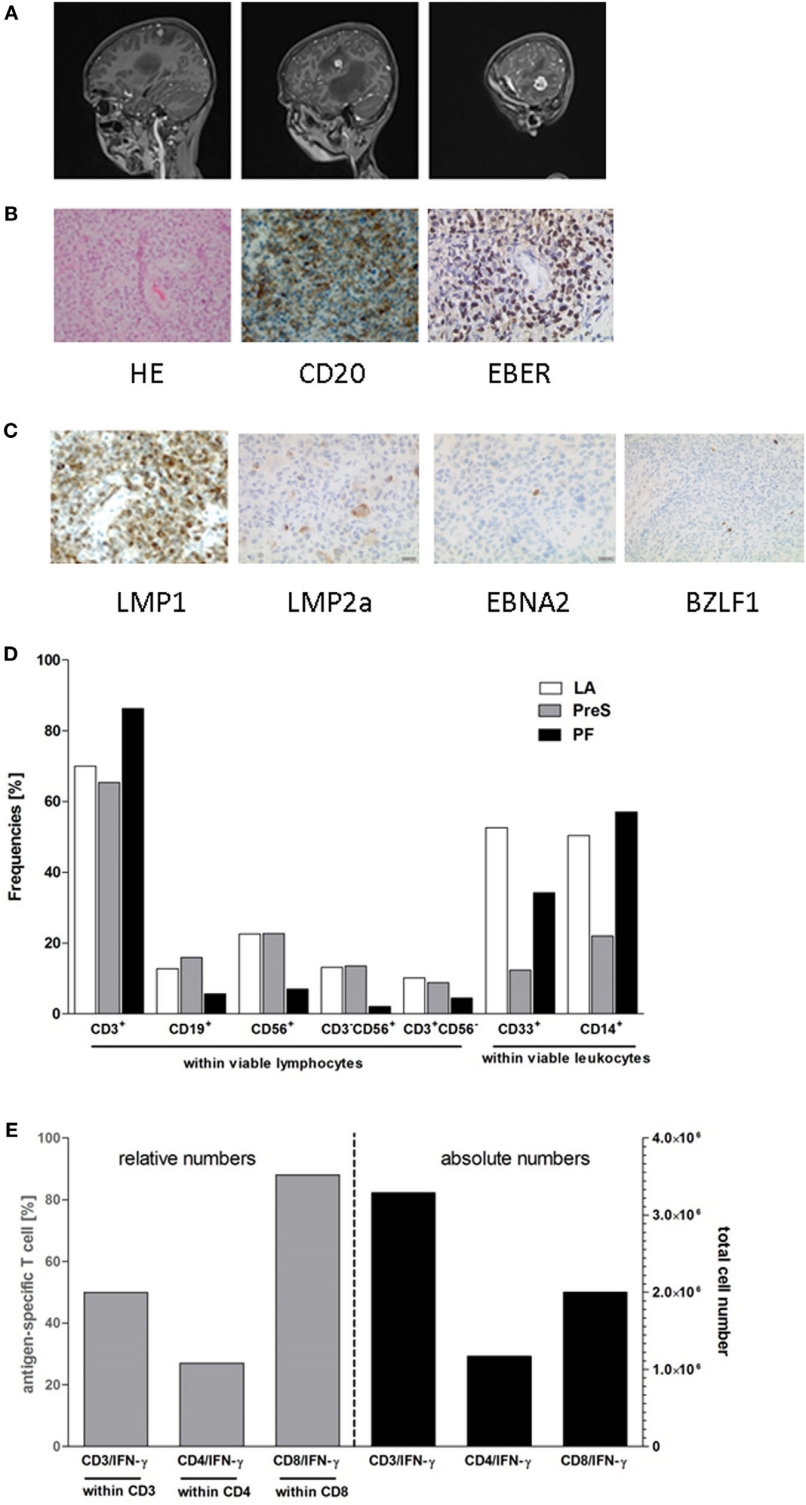
Posttransplant lymphoproliferative disease (PTLD) characteristics and composition of third party donor Epstein–Barr virus (EBV)-specific T-cell product. TPD-derived EBV-CTLs were manufactured by the clinical-scale IFN-γ-based CliniMACS cytokine capture system (CCS) and used for adoptive T-cell transfer (ACT). **(A)** Contrast-enhanced sagittal T1-weighted magnetic resonance imaging images of the patient’s central nervous system at diagnosis of PTLD. Images demonstrate multifocal hyperintense lesions in the left hemisphere in temporal, insular, and parietal lobe. **(B)** Histology of a brain lesion biopsy with staining for H&E and CD20. EBV-association was proven by EBER *in situ* hybridization. **(C)** Expression of EBV products in the lymphoma. LMP1, LMP2a, EBNA2, and BZLF1 were stained by immunohistochemistry. **(D,E)** Composition of the EBV-specific T-cell graft. Proportion of leukocyte subsets and the percentage of IFN-γ secreting EBV-specific T cells were detected after 4 h of *ex vivo* stimulation with the GMP-grade peptide pools EBV ppEBNA1 and ppSelect by flow cytometry. **(D)** Fractions collected during the EBV-specific T-cell manufacturing process [leukapheresis (LA), preselection (PreS), and positive fraction (PF)] were assessed for the proportion of lymphocyte and leukocyte subsets including: CD3^+^ T-cells, CD19^+^ B cells, CD56^+^ NK cells, CD3^+^CD56^+^ NKT cells, CD3^−^CD56^+^ NK cells, CD33^+^ granulocytes, and CD14^+^ monocytes. The compositions of the different cell subsets in the fractions LA, PreS, and PFs are shown. **(E)** The frequencies (left *y*-axis) and numbers (right *y*-axis) of IFN-γ^+^ cells (×10^6^) within the CD3, CD4, and CD8 T-cell populations were analyzed in the PF of the CliniMACS CCS enrichment process to determine the efficiency of the process.

An 11-year-old boy with Alagille syndrome received a related liver allograft during first year of life. Being EBV-negative at transplantation, seroconversion occurred 2 years later. Initial immunosuppression was based on tacrolimus, followed by a combination with mycophenolate mofetil. Ten years after transplantation, he suffered from severe headache, nausea, vomiting, and phono-/photophobia without B symptoms. Funduscopic examination revealed bilateral papilledema. Magnetic resonance imaging (MRI) studies of the brain demonstrated multifocal lesions in the left hemisphere (Figure 1A). After initial treatment for suspected toxoplasmosis, biopsy of the lesion revealed a monomorphic EBV-associated PTLD with features of a diffuse large B-cell lymphoma without *MYC* translocation (Figure 1B). Immunohistochemistry showed expression of CD20 and CD30. Most lymphoma cells expressed EBERs (Epstein–Barr encoded RNAs), LMP1 (EBV latent membrane protein 1), and LMP2a while EBNA2 (Epstein–Barr nuclear antigen 2) and BZLF1 (EBV immediate-early protein) were detected in a low number of neoplastic cells (Figure 1C). EBV PCR was negative in cerebrospinal fluid and weakly positive in peripheral blood (<1,000 copies/ml). Therefore, the diagnosis of EBV-related primary CNS PTLD was made.

Total body imaging and bone marrow aspirate histology displayed no evidence for systemic disease. During initial treatment with dexamethasone, symptoms rapidly improved. Immunosuppression was stopped and immune-/chemotherapy was initiated with six doses of intravenous (i.v.) rituximab (375 mg/m^2^) and weekly intrathecal (i.th.) therapy with rituximab (40 mg), methotrexate (12 mg), cytarabine (30 mg), and prednisone (10 mg) over 10 weeks (9). A partial response by MRI was observed after 3 weeks evolving to complete remission at the end of immuno-/chemotherapy. Due to poor prognosis and the lack of EBV-specific T cells in the patient’s peripheral blood, we decided to consolidate treatment by transfer of partially HLA-matched EBV-CTLs.

## Results and Discussion

The patient received five doses of 2.5 × 10^4^ EBV-CTLs/kg body weight from a 5/10 HLA-matched third party donor (TPD; Table 1). During the production process, CD3+ T-cells were enriched to >80% in the T-cell product with a predominance of CD8+ T-cells (Figures 1D,E; Data Sheet S1 in Supplemental Material). T-cells were administered every 3 weeks in the absence of graft-versus-host disease. After the second injection, the patient developed a skin rash around the neck, which turned out to be atopic dermatitis on histology and responded well to topical steroids without recurrence after subsequent T-cell injections. No other acute or chronic side effects were observed. EBV-PCR remained negative in peripheral blood throughout the whole course. After the end of treatment, immunosuppression was re-introduced with everolimus. At the last follow-up, 2 years after end of cellular therapy, the patient is in continuous remission of PTLD with good organ graft function.

**Figure 2:**
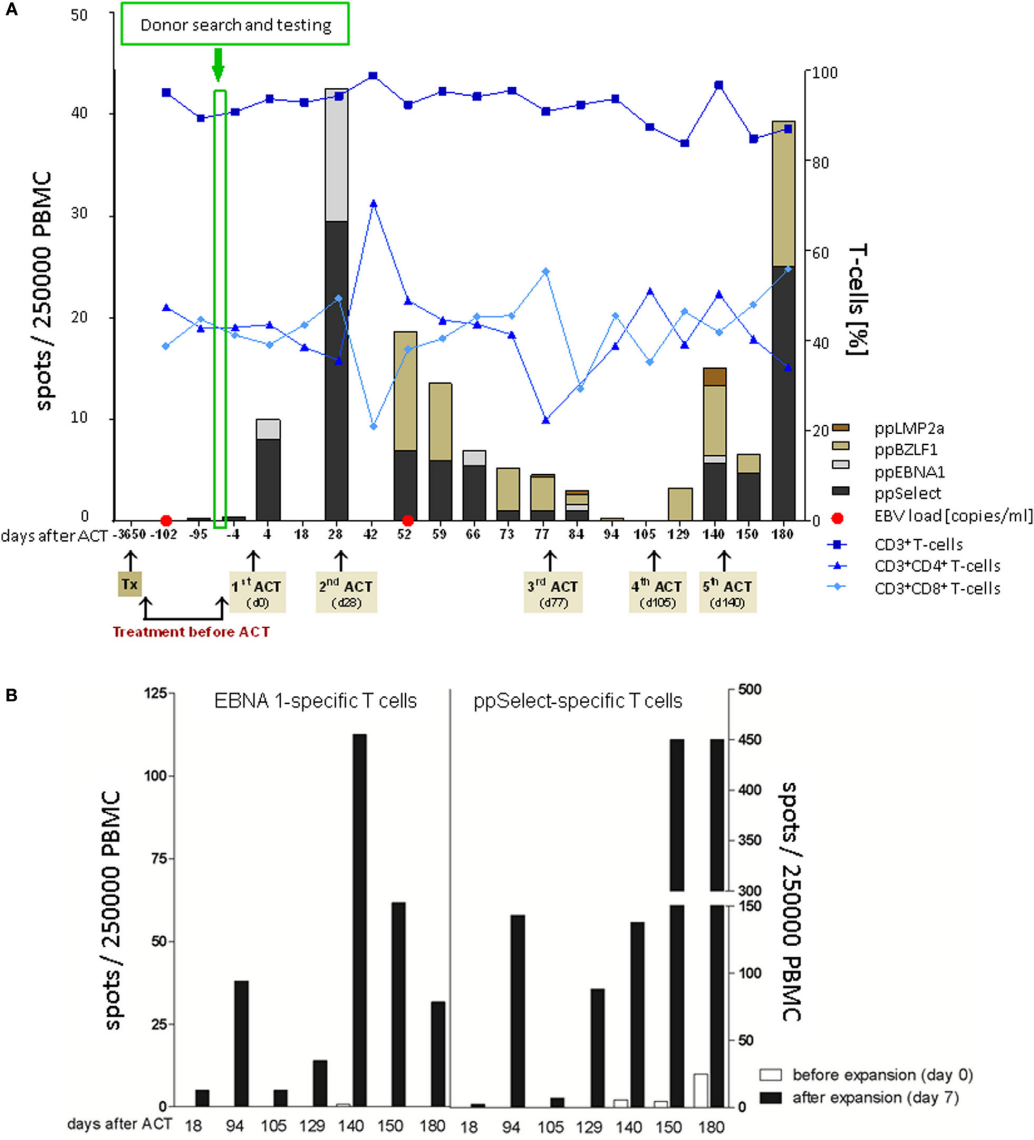
Adoptive T-cell therapy and patient follow-up. **(A)** Monitoring of patients’ cellular immunity was performed with blood samples collected at different time points before and after adoptive T-cell transfer (ACT). Frequencies of CD3, CD4, and CD8 T-cells were assessed by flow cytometry following detection of the Epstein–Barr virus (EBV)-specific T-cell (EBV-CTL) repertoire in response to ppEBNA1, ppSelect, ppBZLF1, and ppLMP2a by using IFN-γ EliSpot. EBV copy numbers were determined in blood and stool samples by quantitative PCR. **(B)** *Ex vivo* expansion of EBV-CTLs. PBMCs were isolated at different time points after ACT [white bars (before expansion, day 0)] and restimulated with the premium-grade peptide pools ppEBNA1 or ppSelect over 7 days [black bars (after expansion, day 7)] followed by the assessment of the EBV-CTL response against ppEBNA1 and ppSelect by IFN-γ Elispot.

No EBV-CTLs were detectable in patient blood on two occasions before adoptive immunotherapy (Figure 2A). In contrast, EBV-CTLs against ppEBNA1 and ppSelect became immediately and constantly detectable 4 days after the first T-cell transfer. While total numbers of CD3+, CD4+, and CD8+ T-cells remained stable throughout the treatment course, EBV-CTLs increased to a maximum of 40 per 250,000 PBMC before the second adoptive transfer. Over time, the target antigens of T-cell response broadened from initially EBNA1 and ppSelect to a broader response including T-cells against LMP2a and BZLF1, respectively (Figure 2A). Since epitopes from these two proteins matching the patient’s or donor’s HLA-type are not contained in the peptide pools used for manufacturing, this suggests that transfer of EBV-specific TPD cells induced an endogenous EBV-directed immune response in the patient, which was absent prior to immunotherapy. Frequency of EBV-CTLs increased during a 7-day *in vitro* restimulation and expansion demonstrating proliferative capacity (Figure 2B).

**Figure 3:**
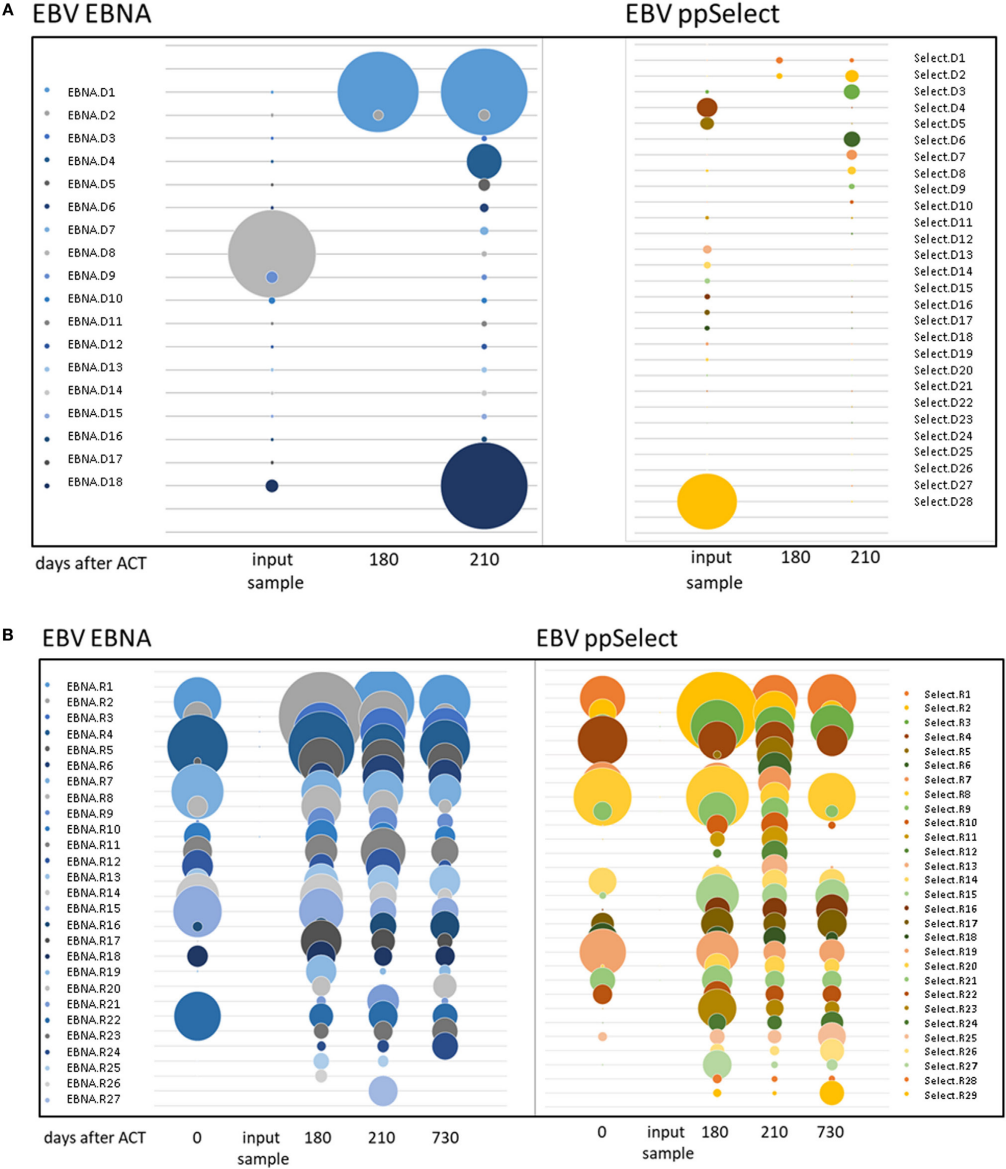
TCR beta chain sequencing of Epstein–Barr virus-stimulated T-cells before and after adoptive transfer. TCR beta chain sequencing was performed on blood samples at different timepoints before and after adoptive T-cell transfer and on the input sample itself. The left panel shows the samples enriched by stimulation with the ppEBNA1 peptide pool, whereas the right panel shows the ones after stimulation with ppSelect. Expansion of different shared clones is shown in both panels for exogenous **(A)** and endogenous **(B)** origin. Clones are labeled according to the antigen, origin (D, donor; R, recipient) and number. TCR sequences can be found in Table S1 in Supplementary Material.

Occasionally, transferred cells could be detected in patient material after transfer, but most authors were unable to retrieve TPD cells on analysis (14). We aimed at dissecting EBV-directed T-cell responses in the T-cell graft and the patient on a clonal molecular level. We performed TCR beta chain (TRB) repertoire analyses by NGS to follow-up the transferred cells and to monitor their expansion to EBV-associated antigens. Investigating the 77 shared clonotypes 41 were identified as expanding clones in CD8+ T cells after the transfer (Figures 3A,B). Four clones could be detected in both follow-up samples at 6 and 7 months after T-cell transfer, while the remaining 37 clones were picked up only once. Notably, the most abundant clone (EBNA.D8 = CASSAGPATNEKLFF, Figure 3A; Table 2) in the enriched T-cell product was not recovered at high abundance while two other clones that made up only 0.001% each of the donor’s CD8 + TRB sequences appeared to expand to 0.51 and 0.17% in two patient samples obtained 7 months after transfer (EBNA.D1 = CASSSKRQVPDTQYF; Select.D6 = CASSPVRSSETQYF, Figure 3A and Table 2). These findings suggest that at least a fraction of the transferred TPD T-cells were expanding and presumably contributing to EBV-specific T-cell responses in the patient. At the same time, we observed a sustained EBNA1-specific expansion of endogenous TRB sequences that were already present in the recipient’s CD8+ T-cell pool before TPD T-cell treatment (Figure 3B). This is consistent with the idea that exogenous T-cells stimulated an efficient endogenous anti-EBV T-cell response and may explain the finding that EBV-T-cell responses against unrelated antigens (LMP2, BZLF1) newly arise after T-cell transfer. Due to limited material availability, we performed the analyses on expanded cells after one *in vitro* peptide pool restimulation, which leaves the possibility of *ex vivo* TCR skewing. These limitations need to be considered in future clinical trials.

Prognosis of CNS PTLD is very poor with 30% overall survival (7, 8). We and others have reported successful administration of intrathecal rituximab; however, efficacy has not been validated in larger series (9, 15). Several studies and case reports show an effect of adoptive T-cell transfer in PTLD (10, 16–19). In particular, patients with CNS PTLD with poor outcome may benefit from this new treatment strategy (8, 9). Haque and colleagues reported responses in 3/5 patients with CNS PTLD after SOT using *in vitro* expanded EBV-specific TPD T-cell lines and lymphoma regression in CNS B-cell lymphoma in an immunodeficiency patient (10, 20). The efficacy of directly isolated EBV-CTLs in CNS PTLD after SOT is still unknown. Studies from patients after stem cell transplantation indicate that these cells are effective in CNS PTLD (19). In the case reported here, combined therapy with intrathecal chemotherapy and rituximab led to sustained complete remission of CNS PTLD. Transfer of partially HLA-matched EBV-CTLs provoked a robust anti-EBV T-cell response containing both exogenous and endogenous TRB signatures; the contribution of T-cell induction to ongoing remission remains uncertain.

Partially HLA-matched TPDs are an attractive source of virus-specific T-cells readily available if pre-screened and registered in T-cell donor registries (13). We did not observe any side effects of TPD T-cell transfer similar to other studies employing virus-specific T-cell therapy, which supports their feasibility and safety. Prospective studies are warranted to prove safety and efficacy of freshly isolated EBV-CTLs from TPDs in this vulnerable patient population.

## Ethics Statement

This case study was carried out in accordance with the Declaration of Helsinki. Treatment was provided on a compassionate use basis. The monitoring protocol was approved by the “ethics committee of Hannover Medical School.” Patient and legal representatives gave written informed consent to the diagnostic program.

## Author Contributions

BM-K and BE-V designed research. RS-F, ST, LK, SR, and CS-F performed research. PH, LG, RB, UK, H-GH, and BE-V manufactured cell product and treated the patient. AK and IA performed histological analysis. ST, LK, SR, CK, IP, BE-V, and BM-K analyzed and interpreted data. RS-F, ST, LK, IP, PH, BE-V, and BM-K wrote the manuscript. All authors read the manuscript and approved the final version.

## Conflict of Interest Statement

The authors declare that the research was conducted in the absence of any commercial or financial relationships that could be construed as a potential conflict of interest.
