# Supplementary material for: Dissecting Epstein-Barr Virus-Specific T-Cell Responses After Allogeneic EBV-Specific T-Cell Transfer for Central Nervous System Posttransplant Lymphoproliferative Disease
Source: Front Immunol. 2018 Jun 27;9:1475. doi: 10.3389/fimmu.2018.01475 (PMC6030255; doi:10.3389/fimmu.2018.01475)
Supplement: Supplementary file 1 [file data_sheet_1.docx]

**Supplemental methods: Details on EBV-specific T cell production**

Manufacturing of EBV-specific T cells was carried out with the CliniMACS Plus device and the MACS GMP PepTivator® EBV_EBNA1 + EBV_Select for antigenic restimulation. Enrichment of IFN-γ-secreting cells was performed by immunomagnetic separation by antibody-conjugated super-paramagnetic particles (CliniMACS IFN-γ Enrichment Reagent, Miltenyi Biotec). Target and non-target cells were quantified by a newly developed single-platform assessment and gating strategy using positive (CD3/CD4/CD8/CD45/IFN-γ), negative (CD14/CD19/CD56), and dead cell (7-AAD) discriminators. The final T cell product had a viability of 73.5% in which 49.7% were EBV-specific IFN-γ positive T cells. For cryopreservation the eluate fraction was adjusted to 2.86% HSA, 7.5% DMSO (dimethyl sulfoxide), aliquoted, subsequently processed in a controlled-rate freezer, and finally transferred to -140 °C or lower in the vapour phase above liquid nitrogen for long-term storage. A fully automated microbial detection system was used for microbiological testing (sterility) of the leukapheresis and the CliniMACS CCS T-cell fraction. Quality control (QC) of the cryopreserved T-cell products (n=4) were performed as described.

**Epstein-Barr virus (EBV)-specific donor T cells**

*Ex vivo* donor cells

Frequency of EBV-specific, IFN-γ^+^ T cells^a^ 1.25% of CD3^+^ T cells

0.44% of CD4^+^ T cells

2.48% of CD8^+^ T cells

Preselected T cells (prior to enrichment)

Viability 96.3%

Frequency of EBV-specific, IFN-γ^+^ T cells 0.88% of CD3^+^ T cells

0.37% of CD4^+^ T cells

1.60% of CD8^+^ T cells

Final T cell product (positive fraction after enrichment)

Viability 73.5%

Lymphocytes (in a volume of 38 ml) 6.4x10^6^ of viable CD45^+^ lymphocytes

95.2% viable CD3^+^ T cells

6.1x10^6^ of viable CD3^+^ T cells

1.9 CD4/CD8 ratio (viable CD3)

Lymphocytes per kg body weight 2.4x10^5^ of viable CD45^+^ lymphocytes 2.3x10^5^ of CD3^+^ T cells

1.1x10^5^ of viable CD3^+^/IFN-γ^+^ T cells

0.4x10^5^ of viable CD4^+^/IFN-γ^+^ T cells

0.7x10^5^ of viable CD8^+^/IFN-γ^+^ T cells

Frequency of EBV-specific, IFN-γ^+^ T cells 49.7% of CD3^+^ T cells

27.4% of CD4^+^ T cells

88.1% of CD8^+^ T cells

Frequency of contaminating lymphocytes 3.08% CD3^-^/CD19^+^ B cells

3.75% CD3^-^/CD56^+^ NK cells

^a^The frequency was determined by cytokine secretion assay in response to the EBV_EBNA1 + EBV_Select peptide pools. The respective negative control has been subtracted from the EBV_EBNA1+Select specific response.
